# Bullet-shaped magnetosomes and metagenomic-based magnetosome gene profiles in a deep-sea hydrothermal vent chimney

**DOI:** 10.3389/fmicb.2023.1174899

**Published:** 2023-06-27

**Authors:** Shinsaku Nakano, Hitoshi Furutani, Shingo Kato, Mariko Kouduka, Toshitsugu Yamazaki, Yohey Suzuki

**Affiliations:** ^1^Graduate School of Science, The University of Tokyo, Tokyo, Japan; ^2^Japan Collection of Microorganisms, RIKEN BioResource Research Center, Tsukuba, Ibaraki, Japan; ^3^Atmosphere and Ocean Research Institute, The University of Tokyo, Chiba, Japan

**Keywords:** magnetotactic bacteria, *Nitrospinae*-related sequences, magnetosome gene cluster, redox gradient, electron microscopy

## Abstract

Magnetosome-producing microorganisms can sense and move toward the redox gradient and have been extensively studied in terrestrial and shallow marine sediment environments. However, given the difficulty of sampling, magnetotactic bacteria (MTB) are poorly explored in deep-sea hydrothermal fields. In this study, a deep-sea hydrothermal vent chimney from the Southern Mariana Trough was collected using a remotely operated submersible. The mineralogical and geochemical characterization of the vent chimney sample showed an internal iron redox gradient. Additionally, the electron microscopy of particles collected by magnetic separation from the chimney sample revealed MTB cells with bullet-shaped magnetosomes, and there were minor occurrences of cuboctahedral and hexagonal prismatic magnetosomes. Genome-resolved metagenomic analysis was performed to identify microorganisms that formed magnetosomes. A metagenome-assembled genome (MAG) affiliated with *Nitrospinae* had magnetosome genes such as *mamA, mamI, mamM, mamP*, and *mamQ*. Furthermore, a diagnostic feature of MTB genomes, such as magnetosome gene clusters (MGCs), including *mamA, mamP*, and *mamQ*, was also confirmed in the *Nitrospinae*-affiliated MAG. Two lines of evidence support the occurrence of MTB in a deep-sea, inactive hydrothermal vent environment.

## Introduction

Magnetotactic bacteria (MTB) produce membrane-enveloped single-domain magnetite (Fe_3_O_4_), greigite (Fe_3_S_4_), or both, which are called magnetosomes (Blakemore, 1975). MTB are phylogenetically affiliated within six major lineages: the *Alpha*-, *Gamma*-, and *Candidatus* (*Ca*.) Etaproteobacteria classes of the *Proteobacteria* phylum, the *Desulfobacterota* phylum, the *Nitrospirae* phylum, and the *Ca*. Omnitrophica phylum (Lefèvre and Bazylinski, 2013; Lin et al., 2014a). Although the morphological features of magnetosomes are different among taxonomic groups, all magnetosomes function as a compass needle for moving along the Earth's geomagnetic field (Frankel et al., 1997). This phenomenon is called magnetotaxis, which enables MTB to inhabit the redox gradient (Frankel et al., 1997; Lefèvre and Bazylinski, 2013). MTB have access to various electron donors and acceptors along the redox gradient. Most of the major lineages use reduced sulfur compounds as energy sources, such as hydrogen sulfide, thiosulfate, and sulfite, whereas O_2_, sulfate, and fumarate are utilized as electron acceptors (Lefèvre et al., 2013; Goswami et al., 2022). Carbon fixation is mediated by *Proteobacteria, Desulfobacterota*, and *Nitrospirae* via the Calvin-Benson cycle or Wood-Ljungdahl (Lefèvre et al., 2013; Goswami et al., 2022). As a result, MTB play an important role in the biogeochemical cycling of iron, sulfur, carbon, and other redox-sensitive elements (Li et al., 2020). Recent studies have reported that MTB are involved in the intracellular deposition of silica (Li et al., 2022), poly-β-hydroxybutyrate (Li et al., 2021), and polymetaphosphate (Schulz-Vogt et al., 2019).

MTB from shallow marine and land environments have been intensively studied for sampling feasibility (Lin et al., 2014a, 2017). Light and electron microscopy was used to observe magnetically separated MTB cells, the taxonomic affiliations of which were determined using fluorescence *in situ* hybridization (FISH) targeting cells containing single-domain magnets (Li et al., 2017). Single-cell genomics and metagenomics combined with light and electron microscopy have been applied to identify MTB by examining the presence of magnetosome gene clusters (MGCs; Kolinko et al., 2012, 2013; Lin et al., 2014b, 2018, 2020). MGCs are consecutively arranged gene sets in MTB genomes that control magnetosome biogenesis (Grünberg et al., 2001; Uebe and Schüler, 2016; McCausland and Komeili, 2020). MGCs have recently been discovered in metagenome-assembled genomes (MAGs) that have been taxonomically classified into previously unknown bacterial lineages for MTB, such as *Nitrospinae, Ca. Latescibacteria, Planctomycetes, Fibrobacteres*, and *Ca. Riflebacteria* (Lin et al., 2020; Uzun et al., 2020).

In contrast, MTB in deep-sea environments is largely unknown, partly because the low cell density of MTB hinders the magnetic recovery of magnetotactic cells sufficiently for FISH and single-cell genomics. In deep-sea sediments, magnetosomes have been observed with microbial cells (Liu et al., 2017; McGlynn et al., 2018; Cui et al., 2021), whereas magnetosomes have been observed without microbial cells (Dong et al., 2016; Yamazaki et al., 2019). Although the previous deep-sea studies attempted to clarify the taxonomy of MTB involved in the formation of magnetosomes by 16S rRNA gene sequences, metagenomic analysis was performed to characterize MTB, the presence of which has been demonstrated by electron microscopy in deep-sea hydrothermal sediments (Chen et al., 2022). However, their metagenomic approach was unsuccessful in revealing the taxonomic affiliations of magnetosome genes and their arrangement into MGCs. It has been demonstrated by our previous studies of deep-sea hydrothermal vent chimneys that chimney samples with high cell densities are predominantly colonized by microorganisms closely related to known MTB lineages (Suzuki et al., 2004; Kato et al., 2010, 2018; Takamiya et al., 2022). In this study, magnetic separation and electron microscopy analyses were performed to detect MTB cells in a metal sulfide chimney sample from the South Mariana Trough (SMT). A genome-resolved metagenomic analysis was also performed to search for MGCs in metagenome-assembled genomes (MAGs) from the vent chimney community.

## Materials and methods

### The site, sample descriptions, and handling

A metal sulfide chimney examined in this study was described in previous research (Kato et al., 2019). The chimney was collected from a hydrothermal vent field at the Pika site (12°55.130′N, 143°38.972′E) in SMT, a back-arc basin where the Philippine Sea Plate is subducted. Sampling was conducted during the Japan Agency for Marine-Earth Science and Technology (JAMSEC) Scientific Cruises NT12-24 of the R/V *Natsushima* in September 2012. The sample size was at a water depth of 2,787 m, and the *in situ* temperature of the deep seawater was 1.7°C. No fluid emanating from the chimney was observed on the sample during video observations. The chimney structure unassociated with fluid venting was collected by the manipulator arm of the remotely operated vehicle, *Hyper Dolphin* (HPD). The chimney structure was enclosed in an HPD container to minimize contamination from the surrounding seawater during transportation to the sea surface.

**Figure 1:**
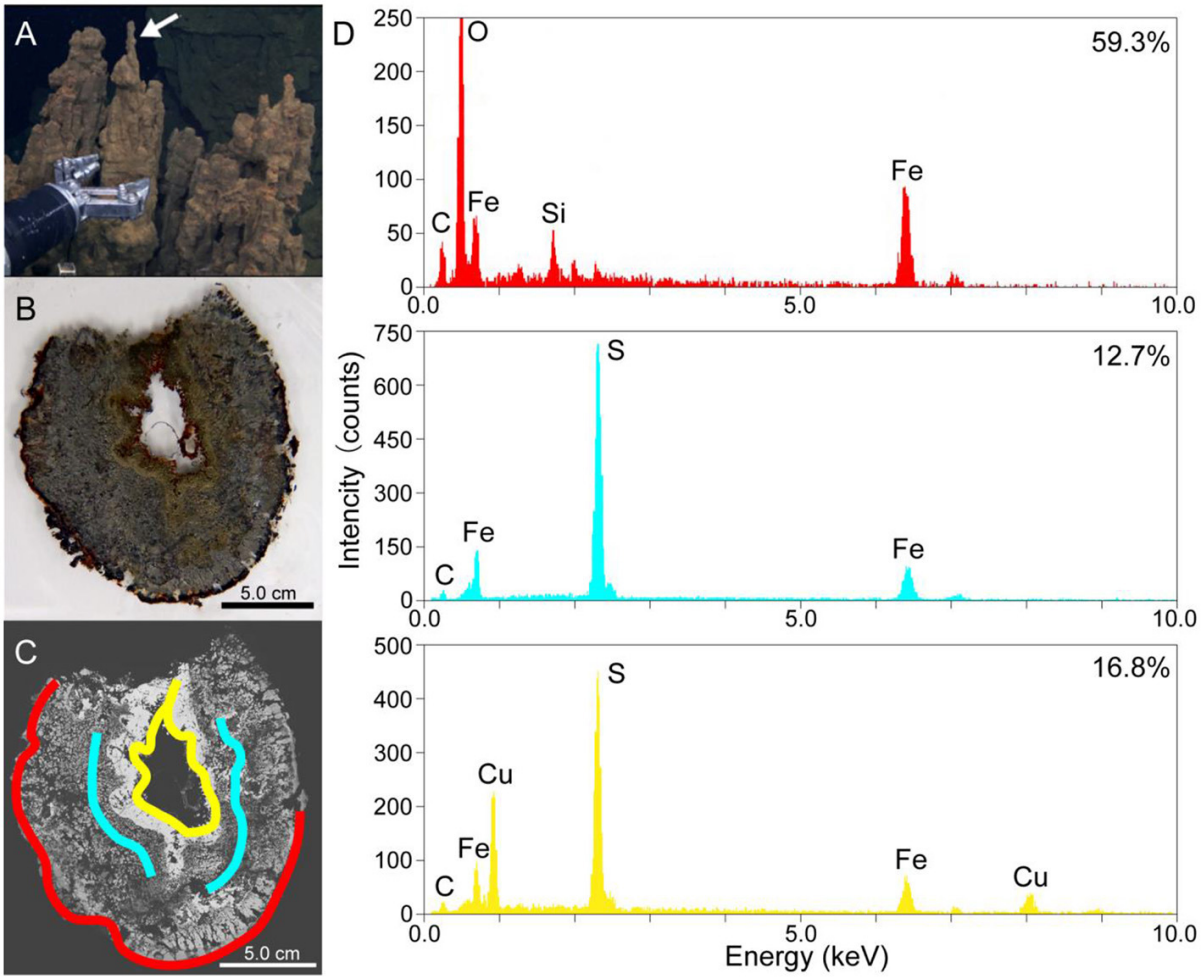
**(A)** A photo of the metal sulfide chimney collected from the Pika site (Pika55). An arrow shows the site where thin sections were made. **(B)** A photo of the thin horizontal section of Pika55. **(C)** A back-scattered electron image obtained by scanning electron microscopy of the thin section of Pika55. Colored bands show the areas drilled for the quantification of amorphous ferric iron. **(D)** Energy-dispersive x-ray spectroscopy spectra from the colored bands in **(C)**. The percentages shown with the spectra indicate the amount of amorphous ferric iron in total iron.

After the retrieval, the chimney structure was named Pika55, which was also described as IPdc by Kato et al. (2019) and was immediately subsampled onboard in a cold room at 4°C. First, the tip of the metal sulfide chimney sample (shown by a white arrow in Figure 1A) was soaked two times in 100% ethanol for 5 min to dehydrate, and the chimney sample was infiltrated four times with LR White Resin for 30 min and solidified in an oven at 50°C for 48 h. Solidified blocks were cut into thin sections and polished with corundum powder and diamond paste. Then, after the removal of the chimney tip, the interior and exterior portions of the chimney sample were separated using sterile chisels and spatulas. The exterior portion was named Pika55ext. Subsequently, Pika55ext was ground into powder using a sterile pestle and mortar and stored at −80°C for magnetic separation and metagenomic analysis. Finally, some ground samples were fixed with 3.7% formamide in seawater onboard for magnetic separation.

### Magnetic separation and electron microscopy

Magnetic particles were separated from the Pika55ext ground sample by sonicated dispersion in a sodium hexametaphosphate buffer solution, followed by sample collection by Nd magnets. The extracted magnetic particles dispersed in ethanol were mounted on a carbon-coated copper grid. A JEM-1400 transmission electron microscope (TEM; JEOL, Tokyo, Japan) was used to observe magnetic particles at an operation voltage of 120 kV. The extracted magnetic particles were prefiltered through 8.0-μm pore size nitrocellulose filters and then filtered through 0.2-μm pore size polycarbonate filters. The filtered magnetic particles were carbon-coated and observed using the S4500 scanning electron microscope (SEM; Hitachi, Ibaragi, Japan) at an accelerating voltage of 10 kV. Finally, secondary electron imaging coupled to energy-dispersive x-ray spectroscopy (EDS) was performed to clarify the chemical compositions of magnetosomes.

### Metagenomic analysis

As described in a previous study, DNA extraction, library construction, and shotgun sequencing were performed (Kato et al., 2019). Using an UltraClean Soil DNA Isolation Kit (MoBio Laboratories, Carlsbad, CA, USA), genomic DNA was extracted from Pika55ext without the use of magnetic separation. Hirai et al. (2017) constructed a shotgun library for the extracted DNA using a KAPA Hyper Prep kit for Illumina (KAPA Biosystems, Wilmington, MA, USA). Library sequencing was performed on an Illumina MiSeq platform (MiSeq PE300). The following steps were taken to reconstruct MAGs using MetaWRAP v.1.3.2 (Uritskiy et al., 2018). First, the Read_QC module included in MetaWRAP was used to trim and filter reads from the library. Following that, high-quality reads were assembled into contigs using SPAdes version 3.13.0 with the options “–meta, -k 55,77,99,111,121” (Bankevich et al., 2012). Then, the contigs were binned into metagenome-assembled genomes (MAGs) using the Binning module [including metabat2 (Kang et al., 2015), maxbin2 (Wu et al., 2016), and concoct (Alneberg et al., 2014)] included in MetaWRAP. The MAGs from the binning tools were then refined using the bin refinement module included in MetaWRAP. For phylogenomic analysis, MAGs with a completeness of >70% and a contamination of < 5% were selected (Supplementary Table 1). The taxonomic classification was performed based on the National Center for Biotechnology Information (NCBI) taxonomy (Sayers et al., 2021) and the Genome Taxonomy Database (GTDB) taxonomy (Parks et al., 2022). Magnetosome genes were searched against the MAGs using the FeGenie program with a lower maximum distance of 1 or 5 (Garber et al., 2020). BLASTP (Gish and States, 1993) was also used to search for magnetosome genes in the MAGs from this study and the NCBI database (Gish and States, 1993). Relative abundances of the MAGs in the metagenome were estimated based on the normalized read coverage values using the Quant Bins module included in MetaWRAP.

### Phylogenetic analysis of magnetosome genes

Known MTB genomes were downloaded from the NCBI database against which magnetosome genes were searched using the FeGenie program (Garber et al., 2020). Then, the amino acid sequences of magnetosome genes were aligned using Muscle v.3.8.8425 (Edgar, 2004) within the ARB software (Ludwig et al., 2004) and filtered using the TrimAl program (Capella-Gutierrez et al., 2009) with the “-gappyout” option. Additionally, maximum-likelihood phylogenetic protein trees were constructed using RAxML v.8.2.11 (Stamatakis, 2014) in the Geneious Prime software. The maximum-likelihood trees were obtained using the 1,000 bootstrap-resampling approaches. Protein trees were visualized using FigTree v.1.4.4 (http://tree.bio.ed.ac.uk/software/figtree/) and were rooted at the midpoint.

Based on 120 concatenated bacterial single-copy marker proteins, a maximum-likelihood tree was constructed for *Nitrospinae* genomes downloaded from the GTDB database (Parks et al., 2017). With the “-gappyout” option, the TrimAl program was used to trim concatenated sequences. The Geneious Prime software's RAxML v.8.2.11 was used to build the maximum-likelihood tree. The maximum-likelihood tree was obtained using the 1,000 bootstrap-resampling approach. The tree was rooted with *Thermodesulfovibrio yellowstonii* DSM 11347 and visualized using FigTree v.1.4.4.

### Metabolic predictions

A curated set of genes involved in carbon, nitrogen, and sulfur metabolism were searched using METABOLIC v.4.0 (Zhou et al., 2022), which annotates genes through the Kyoto Encyclopedia of Genes and Genomes (KEGG; Ogata et al., 1999), TIGRfam (Selengut et al., 2007), Pfam (Finn et al., 2014), and custom hidden Markov model profiles. Using DiSCo, genes involved in sulfur metabolism, such as *dsrABCDEFHJKMOP, aprAB*, and *sat*, were annotated (Neukirchen and Sousa, 2021). KEGG was used to annotate *sir*, which METABOLIC and DiSCo did not annotate. In Supplementary Table 2, the complete names of metabolic genes and the related annotation tools are presented.

## Results and discussion

### Internal redox gradient indicated by amorphous Fe(III) quantification

Thin sections of the metal sulfide chimney collected from SMT called Pika55 were examined to reveal the redox gradient, which is potentially important for magnetotaxis in the chimney. Light microscopy observations showed that the internal structure was concentrated in three layers (Figures 1A, B). The outermost layer was reddish, and a gray layer was present inside the reddish layer. Moreover, the innermost metallic gold layer had a reddish inner rim. The combination of these layers is typical for metal sulfide chimneys found at deep-sea hydrothermal vents (Haymon, 1983). Additionally, SEM observations with back-scattered electron imaging and EDS analysis were performed to show that the chimney mainly comprises iron and sulfur or iron, copper, and sulfur (Figures 1C, D). In the outer layer, a dark contrast phase relative to metal sulfides was composed of Fe, Si, and O (Figures 1C, D). In contrast, the two inner layers were unassociated with the iron silicate phase.

As the iron silicate phase is likely composed of ferric iron due to the oxidative alteration of iron-bearing sulfides, the valence state of iron was clarified by a colorimetric ferrozine-based assay (Lovley and Phillips, 1986) coupled with the micron-scale drilling technique (Sakai and Kodan, 2011). The reddish layer contained 59.3% of amorphous ferric iron in the total iron extracted with 0.5-M HCl (red band and EDS spectrum in Figures 1C, D), whereas the inner and outer portions of the gray layer contained 12.7% (blue bands and EDS spectrum in Figures 1C, D). The innermost metallic layer contained 16.8% amorphous ferric iron in total iron extracted with 0.5-N HCl (yellow band and EDS spectrum in Figures 1C, D). Finally, there appears to be a redox gradient between the outermost and innermost parts of the chimney structure.

### Microbial cells with bullet-shaped magnetosomes found in the metal sulfide chimney

**Figure 2:**
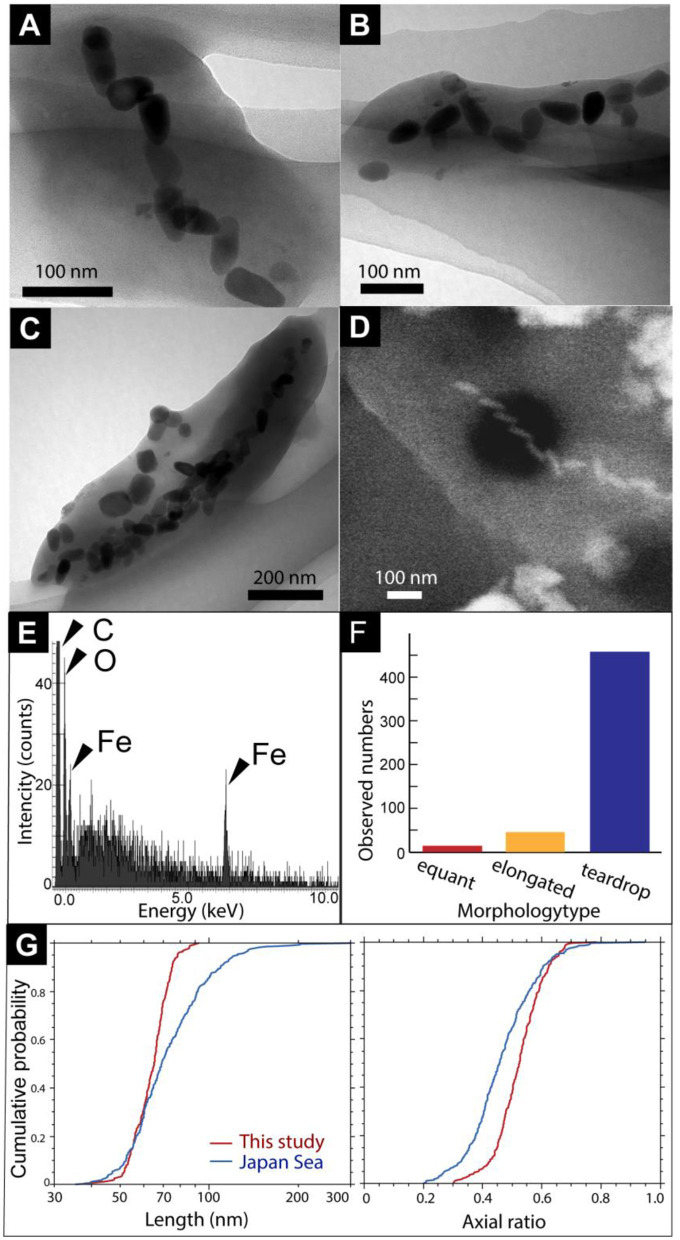
**(A)** A TEM image of a cell with bullet-shaped magnetosomes. **(B, C)** TEM images of magnetosomes unassociated with cells. Black arrows show cubo-octahedral magnetosomes. White arrowheads show hexagonal prismatic magnetosomes. **(D)** Secondary electron image of a microbial cell with bullet-shaped magnetosomes. **(E)** Energy dispersive x-ray spectrum from a red circle in **(D)**. **(F)** Observed a number of morphology types of magnetosomes. **(G)** Cumulative probability distribution of length and axial ratio (width/length) for bullet-shaped crystals extracted from the chimney sample in this study (red) and from Japan Sea surface sediments (blue; Yamazaki, 2020).

First, magnetic separation of the metal sulfide chimney sample from SMT was performed. TEM observations of the magnetically separated particles revealed microbial cells with bullet-shaped magnetosomes (Figures 2A–C). The bullet-shaped magnetosomes in ~200-nm-wide and 500-nm-long microbial cells were arranged into a single chain (Figure 2A). SEM observations and EDS analysis of a microbial cell with bullet-shaped magnetosomes, similarly observed by TEM, revealed that the bullet-shaped magnetosomes were composed of Fe and O without S (Figures 2D, E). The observed morphology types of the magnetosomes without microbial cells were categorized as bullet-shaped, cuboctahedral, and hexagonal prismatic (Figures 2B, C). Enumeration of each morphology type revealed the dominance of bullet-shaped magnetosomes in the chimney sample (Figure 2F). As the composition of cuboctahedral and hexagonal prismatic magnetosomes was not examined, the possibility that these particles were not magnetosomes cannot be ruled out. Based on TEM images, the length and width of bullet-shaped crystals associated with and without microbial cells (*n* = 253) were measured. The median length and axial ratio (width/length) were 65.2 nm and 0.525, respectively (Figure 2G). The distribution of crystal size is significantly uniform, relative to that of bullet-shaped magneto fossils in surface marine sediments from the Japan Sea (Yamazaki, 2020). These results indicate the inhabitation of a limited species of MTB in a metal sulfide chimney.

### Metagenomic evidence of the production of magnetosomes in the chimney by *Nitrospinae*

**Figure 3:**
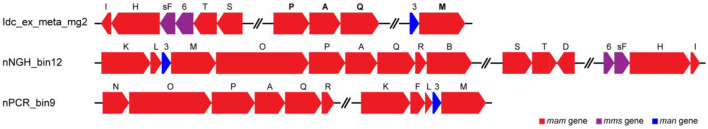
Comparison of magnetosome gene cluster arrangements in *Nitrospinae*-affiliated MAGs from this study (Idc_ex_meta_mg2) and a previous study (Lin et al., 2020). Magnetosome genes annotated by FeGenie, in addition to BLASTP scoring, are indicated in bold font (Table 1 and Supplementary Table 1).

Metagenomic analysis was performed to obtain MAGs from the vent chimney samples (Table 1). Based on reading coverage values, abundant NCBI-based taxonomic groups were *Nitrospirae* (p_Nitrospirota; c_Thermodesulfovibrionia; o_Thermodesulfovibrionales; f_JdFR-85; and g__BMS3Bbin07 based on the GTDB taxonomy) represented by a MAG named Idc_ex_meta_mg7 and *Gammaproteobacteria* (p_Proteobacteria; c_Gammaproteobacteria; o_Arenicellales; and f-s_BMS3Bbin11 based on the GTDB taxonomy) represented by a MAG named Idc_ex_meta_mg1 (Table 1). 16S rRNA gene sequences were not found in all MAGs except for the MAG Idc_ex_meta_mg1. Based on the 16S rRNA gene sequence, the MAG Idc_ex_meta_mg1 had no close MTB relatives. All MAGs were subjected to a FeGenie search against *mamABEIKLMOPQ*. *mamABEIKLMOPQ* were selected because these magnetosome genes are universal for magnetite production by MTB (Kolinko et al., 2016; Uebe and Schüler, 2016). As a result, it was revealed that *mamA, mamI, mamM, mamP*, and *mamQ* were found in a MAG called Idc_ex_meta_mg2 affiliated with *Nitrospinae* (p_Nitrospinota; c-o_UBA7883 based on the GTDB taxonomy). The consecutive arrangement of *mamP, mamA*, and *mamQ* with locus tags of Idc_ex_meta_mg2_1352 to 1354 in the *Nitrospinae-*affiliated MAG supports the presence of an MGC (Supplementary Table 1). Other magnetosome genes previously found in *Nitrospinae*-affiliated MAGs (nPCR_bin9 and nNGH_bin12; Lin et al., 2020) were searched, and the arrangement of the MGC (*mamP, mamA*, and *mamQ*) in Idc_ex_meta_mg2 is similar to those from the other *Nitrospinae*-affiliated MAGs (Figure 3 and Supplementary Table 1). As a result, the presence of two other MGC clusters was due to the composition of *mamI, mamH, mmsF, mms6, mamT*, and *mamS*, and *man3* and *mamM* (Figure 3 and Supplementary Table 1). The former cluster was similar to that found in nNGH_bin12, whereas the latter cluster was similar to that in nPCR_bin9 (Figure 3). It should be noted that the MGCs found in the *Nitrospinae*-affiliated MAGs were similar to those in *Alphaproteobacteria* and *Ca*. Etaprotebacteria but clearly different from those in *Desulfobacteria, Nitrospirae*, and *Ca*. Omnitrophica. Although *mamA* and *mamE* were detected in MAGs other than *Nitrospinae* (non-*Nitrospinae* MAGs) by FeGenie, the detected *mamA* and *mamE* were not consecutively arranged without forming MGCs. Although *mamA* and *mamE* found in non-*Nitrospinae* MAGs may function in magnetosome formation, the functions of *mamA* and *mamE* in non-*Nitrospinae* MAGs need to be clarified.

**Table 1:** List of metagenome-assembled genomes and their genomic features.

**MAG ID**	**Genome size (bp)**	**Numbers of contigs**	**Completeness (%)**	**Contamination (%)**	**Coverage (x)**	**Accession**	**Taxonomy based on NCBI**	**Taxonomy based on GTDB**	**Magnetosome genes identified by FeGenie**
Idc_ex_meta_mg1	2,572,058	165	98.17	2.08	21.19	DRZ059048	p_Proteobacteria; c_Gammaproteobacteria	p_Proteobacteria; c_Gammaproteobacteria; o_Arenicellales; f_BMS3Bbin11; g_BMS3Bbin11; s_BMS3Bbin11 sp002897635	*mamE*
Idc_ex_meta_mg2	2,014,408	355	90.5	3.59	3.41	DRZ059049	p_Nitrospinae; c_Nitrospinia; o_Nitrospinales	p_Nitrospinota; c_UBA7883; o_UBA7883	*mamA, mamI, mamM, mamP, mamQ*
Idc_ex_meta_mg3	1,412,330	531	83.1	3.96	2.74	DRZ059050	p_Proteobacteria; c_Epsilonproteobacteria	p_Campylobacterota; c_Desulfurellia; o_JAADFJ01; f_JAADFJ01; g_JAADFJ01	*mamE*
Idc_ex_meta_mg6	2,753,789	141	97.85	0.67	8.72	DRZ059051	p_Bacteroidetes; c_Bacteroidia; o_Bacteroidales	p_Bacteroidota; c_Bacteroidia; o_Bacteroidales; f_F082; g_SZUA-53	*mamA, mamE*
Idc_ex_meta_mg7	2,256,723	191	97.22	0.91	37.38	DRZ059052	p_Nitrospirae	p_Nitrospirota; c_Thermodesulfovibrionia; o_Thermodesulfovibrionales; f_JdFR-85; g_BMS3Bbin07	*mamA, mamE*
Idc_ex_meta_mg8	3,037,378	1,316	74.38	3.36	2.66	DRZ059053	p_Spirochaetes; c_Spirochaetia	p_Spirochaetota; c_UBA6919; o_UBA6919; f_UBA6919	*mamE*

### Phylogenetic congruency of magnetosome genes in the *Nitrospinae* genome

**Figure 4:**
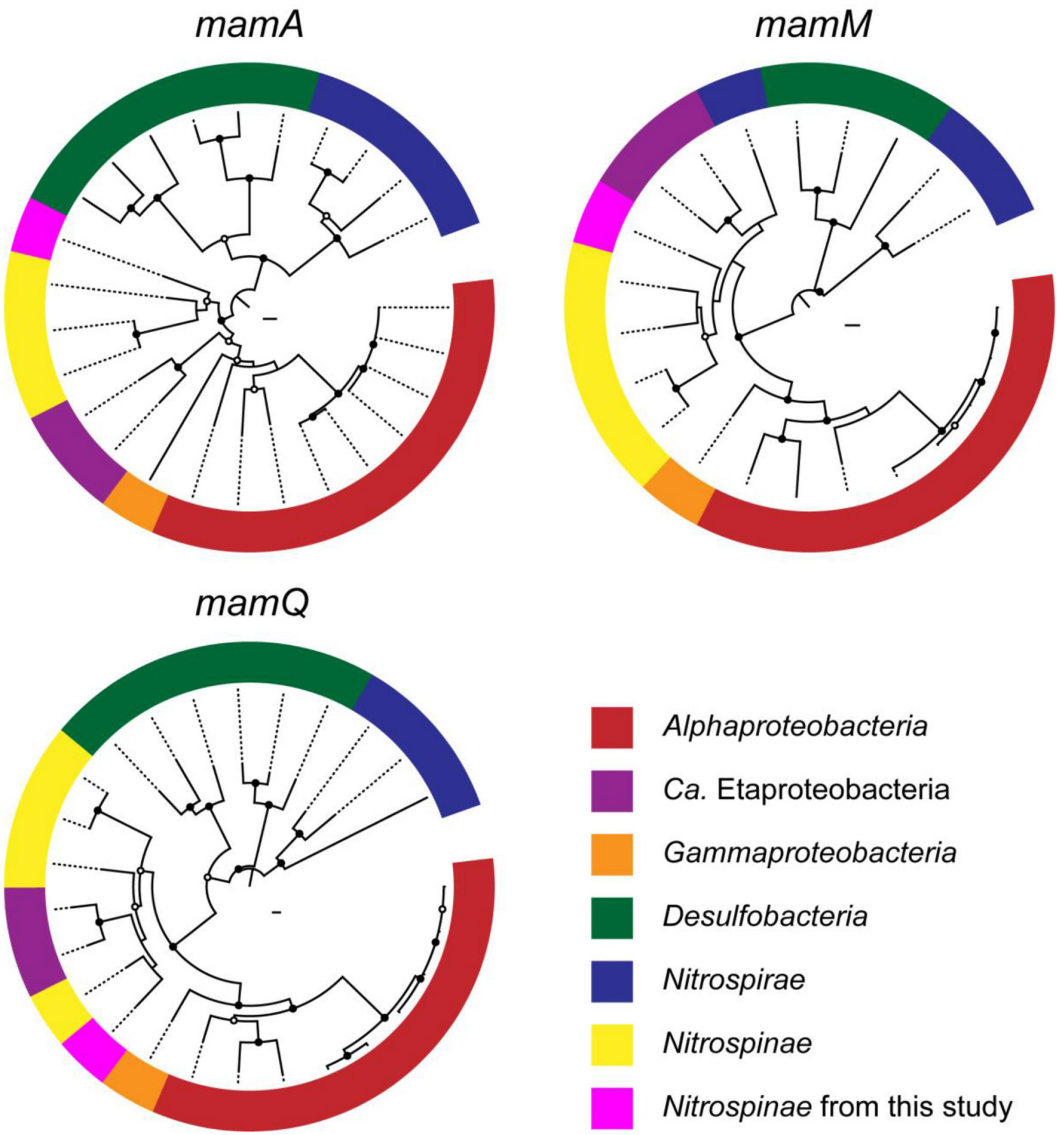
Phylogenetic trees of magnetosome genes, *mamAMQ*. The trees were constructed with the *mam* genes of known MTB (*Proteobacteria, Desulfobacteria*, and *Nitrospirae*) and *Nitrospinae* genomes. Filled and open black circles at nodes represent 1,000 pseudoreplicate bootstrap values higher than 75 and 50%, respectively. Complete trees are shown in Supplementary Figures 1–3.

Phylogenetic trees were constructed for *mamAMQ* found in the *Nitrospinae*-affiliated MAG obtained in this study (Figure 4). *MamAMQ* genes were selected because the gene sequences are present in most MTB genomes with sufficient sequence lengths for phylogenetic analysis (Lin et al., 2020). Consequently, the *mamAMQ* sequences from the chimney *Nitrospinae* formed a monophyletic clade with those from *Nitrospinae*-affiliated genomes from public databases rather than those from the other major taxonomic groups of MTB, except for the *mamQ* gene (Figure 4). The low bootstrap values might have resulted from the genetic divergence of magnetosome genes in *Nitrospinae*. Based on the relatedness of the *Nitrospinae*-affiliated magnetosome genes, the MGC appears to be encoded in the chimney *Nitrospinae* genome. Although the presence of MGC is a prerequisite for MTB, it remains to be determined whether the chimney *Nitrospinae* is capable of producing magnetosomes.

### The metabolic potential of the chimney *Nitrospinae*

**Figure 5:**
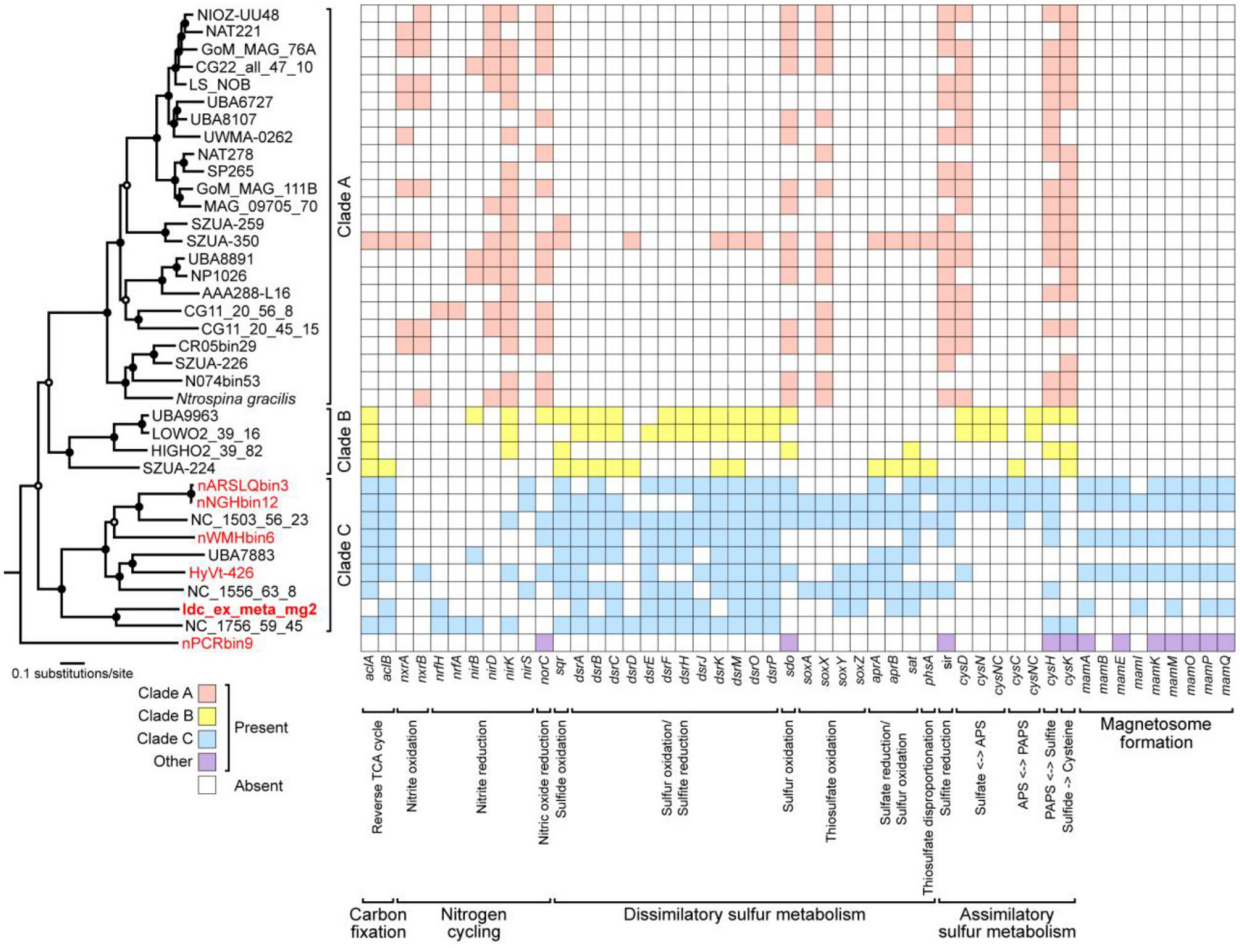
A maximum-likelihood tree of 120 concatenated bacterial single-copy marker proteins from *Nitrospinae* genomes, as well as a heat map of *Nitrospinae* genomes showing the presence or absence of genes involved in carbon, nitrogen, and sulfur metabolisms as well as magnetosome genes. Genes involved in magnetosome formation were annotated by FeGenie. Other genes were annotated by METABOLIC and DiSCo. A concatenated sequence obtained in this study is indicated in bold letters, whereas concatenated sequences containing MGCs are indicated in red letters. At nodes in the tree, filled and open black circles represent 1,000 pseudoreplicate bootstrap values higher than 75 and 50%, respectively. The absence of *aclAB* in Clade A may be due to the low similarity of *aclAB* sequences in Clade A genomes to known *aclAB* sequences.

Genes involved in energy-yielding metabolic pathways were annotated using METABOLIC and DiSCo (Supplementary Table 2). The presence of *nrfH* (locus tag: Idc_ex_meta_mg2_0657) in the chimney *Nitrospinae* genome indicates nitrite reduction as the terminal electron-accepting process (Idc_ex_meta_mg2 in Figure 5). In mediating nitrite reduction, *nrfA* is essentially present at the consecutive downstream of *nrfH*. *nrfA* was found downstream of the *Nitrospinae* genome (locus tag: Idc_ex_meta_mg2_0656), wherein lysine, a motif sequence, was substituted with histidine. This substitution has also been found in a *nrfA*-like gene in *Campylobacter jejuni* (Einsle et al., 2000), whose nitrite-reducing ability has been demonstrated (Pittman et al., 2007). Thus, the chimney *Nitrospinae* may depend on nitrite as the electron acceptor. As for the energy source, sulfur oxidizers have an operon, including *dsrEFH* and a Dsr-Apr-Sat system, typically possessed by sulfate reducers (Dahl et al., 2008). In the *Nitrospinae* genome, *dsrA* (locus tag: Idc_ex_meta_mg2_1291), *dsrE* (locus tag: Idc_ex_meta_mg2_1294), *dsrF* (locus tag: Idc_ex_meta_mg2_1293), *dsrH* (locus tag: Idc_ex_meta_mg2_1292), *sat* (locus tag: Idc_ex_meta_mg2_1547), and *aprB* (locus tag: Idc_ex_meta_mg2_0024) were found (Figure 5). These results indicate the potential that the chimney *Nitrospinae* is a nitrite-reducing sulfur oxidizer.

*Nitrospina gracilis*, a cultivated member of the phylum *Nitrospinae*, is a chemolithoautotroph that generates energy by oxidizing nitrite (Lucker et al., 2013). Draft *Nitrospinae* genomes were submitted to comparative genomic analysis to clarify the variation of metabolic pathways in phylogenetically diverse members of the phylum *Nitrospinae* (Figure 5 and Supplementary Table 3). Three class-level clades were identified using a maximum likelihood tree from the concatenated sequences of 120 bacterial single-copy marker genes. Clade A included *N. gracilis*, whereas Clade B and Clade C included uncultivated taxa. The chimney genome (Idc_ex_meta_mg2) and the other MGC-encoding genomes were found in Clade C. *N. gracilis* uses the reductive tricarboxylic acid (rTCA) cycle for CO_2_ fixation. *Acl* is a key enzyme in the rTCA, and *N. gracilis* has an *aclAB* with low sequence similarities to known *aclABs* (Lucker et al., 2013). The low sequence similarities caused *aclAB* to be undetected by most of the Clade A genomes, including *N. gracilis* by METABOLIC.

In contrast, *aclAB* was detected in many Clade B and C genomes, including the chimney *Nitrospinae*. Thus, the chimney *Nitrospinae* is most likely a chemolithautotroph that uses rTCA for CO_2_ fixation. *nxrAB*, which is involved in nitrite oxidation by *N. gracilis* (Lucker et al., 2013), was present in 11 genomes affiliated with Clade A, but *nxrAB* was absent in 13 genomes affiliated with Clades B and C, except for HyVt-426. *sir*, indicated to be involved in assimilatory sulfite reduction by *N. gracilis* (Lucker et al., 2013), was detected in many genomes from Clade A and Clade C. Sulfur oxidation genes such as *dsrEFH* were not found in Clade A genomes, which is consistent with the use of nitrite as an energy source.

In contrast, many genomes affiliated with Clades B and C contained *dsrEFH*, as well as *aprAB* and *sat*. According to these results, the metabolic potential of the chimney *Nitrospinae* appears to be common in Clade C but is clearly distinct from that of Clade A, which includes *N. gracilis*. Taken together, the bacteria from the phylum *Nitrospinae* play an important role in carbon fixation in dark deep-sea environments.

## Conclusion

In this study, magnetosomes in a metal sulfide chimney were demonstrated through direct observations of magnetically separated cells. Based on the genome-resolved metagenomic analysis, some of these magnetosomes could have originated from *Nitrospinae*-affiliated populations possessing genes involved in magnetosome production. Thus, our results expand the ecological diversity of magnetosome-producing MTB. However, it is still unclear what the ecological advantage of magnetosomes in the metal sulfide chimney is. To answer this question, further culture-dependent and culture-independent analyses are needed.

## Data availability statement

The original contributions presented in the study are included in the article/Supplementary material. The MAG sequences presented in this study can be found in online repositories. The names of the repository/repositories and accession number can be found at: www.ncbi.nlm.nih.gov under the accession number, PRJDB13464.

## Author contributions

SN and YS designed the study and co-wrote the manuscript. YS, HF, and SK collected and analyzed the chimney sample as shipboard scientists during JAMSTEC Scientific Cruises NT12-24. SN, HF, and YS performed mineralogical characterizations. SN and TY conducted magnetic separation and electron microscopy. SN, MK, HF, SK, and YS performed single-gene and metagenomics analyses and data analyses. All authors discussed the results and commented on the manuscript. All authors contributed to the article and approved the submitted version.
