# Supplementary material for: Bullet-shaped magnetosomes and metagenomic-based magnetosome gene profiles in a deep-sea hydrothermal vent chimney
Source: Front Microbiol. 2023 Jun 27;14:1174899. doi: 10.3389/fmicb.2023.1174899 (PMC10335762; doi:10.3389/fmicb.2023.1174899)
Supplement: Supplementary file 2 [file Data_Sheet_1.DOCX]

Supplementary Material

**
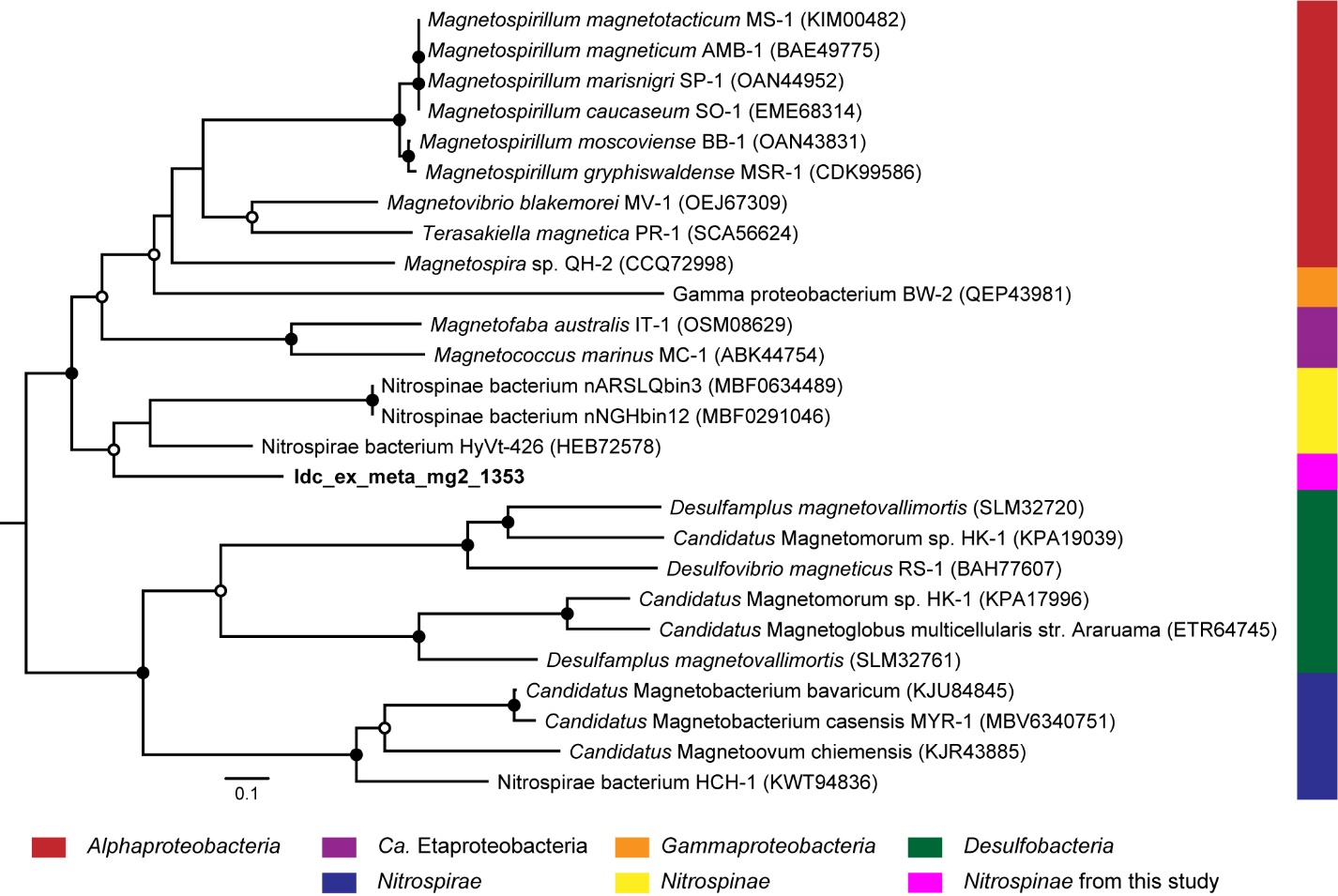
**

**Supplementary Figure 1.** Maximum-likelihood tree of *mamA* gene sequences. The tree was constructed with sequences from this study and from public databases including known MTB and *Nitrospinae*. Sequences obtained in this study are represented by bold characters. Filled and open black circles at nodes represent 1,000 pseudoreplicate bootstrap values higher than 75% and 50%, respectively.

**
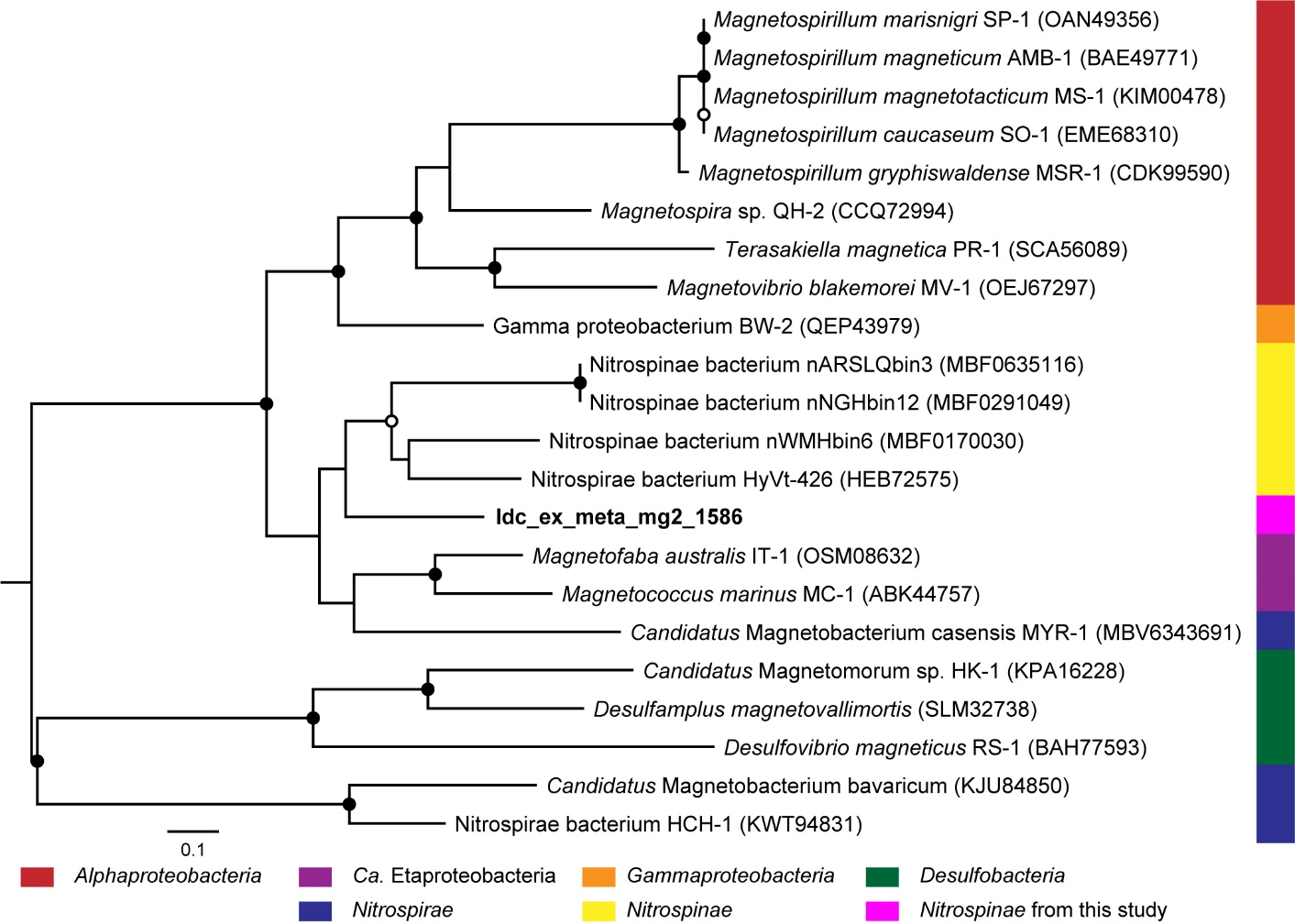
**

**Supplementary Figure 2.** Maximum-likelihood tree of *mamM* gene sequences. The tree was constructed with sequences from this study and from public databases including known MTB and *Nitrospinae*. Sequences obtained in this study are represented by bold characters. Filled and open black circles at nodes represent 1,000 pseudoreplicate bootstrap values higher than 75% and 50%, respectively.

**
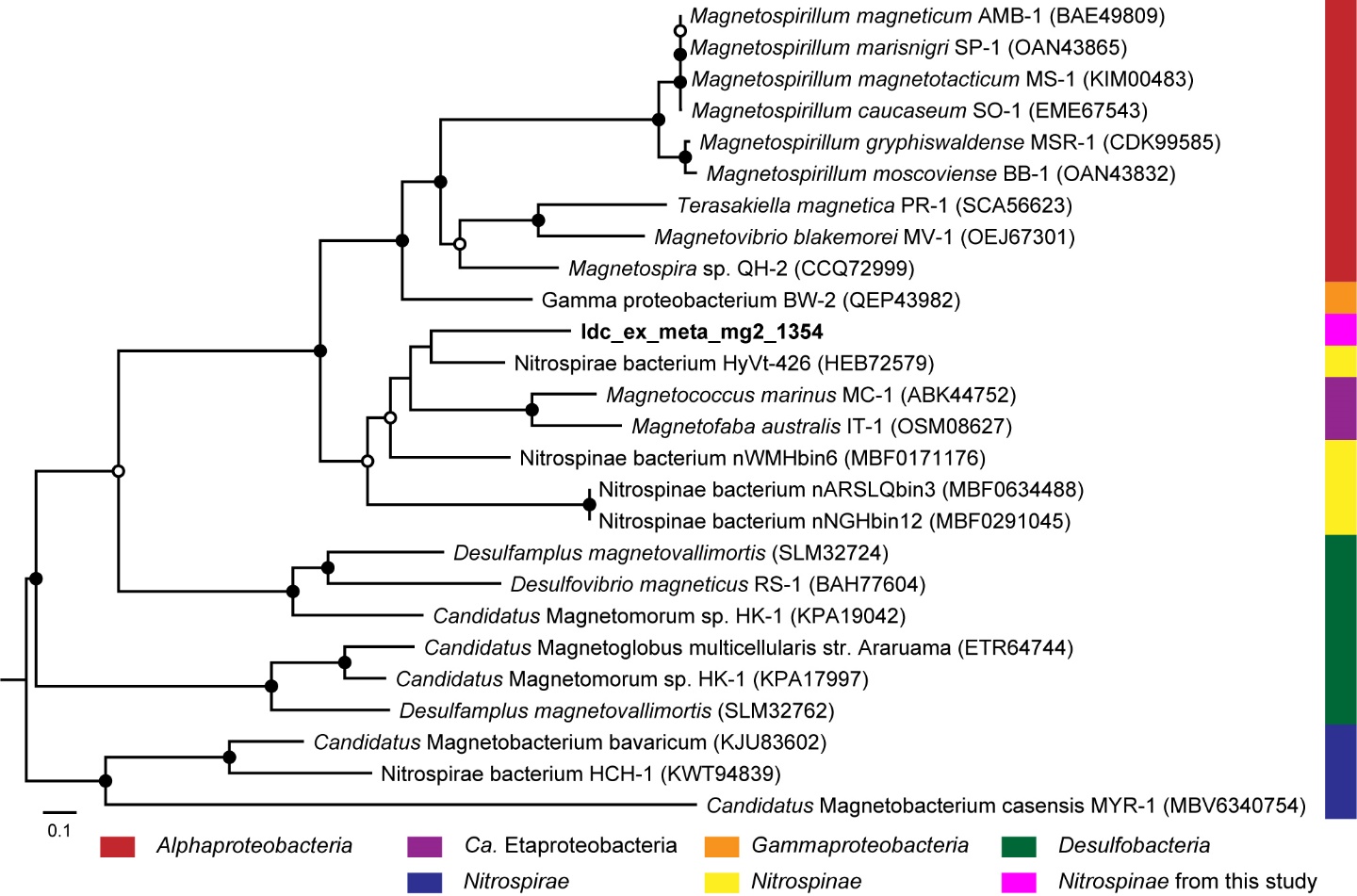
**

**Supplementary Figure 3.** Maximum-likelihood tree of *mamQ* gene sequences. The tree was constructed with sequences from this study and from public databases including known MTB and *Nitrospinae*. Sequences obtained in this study are represented by bold characters. Filled and open black circles at nodes represent 1,000 pseudoreplicate bootstrap values higher than 75% and 50%, respectively.
